# A case report of FAVA syndrome in a young woman carrying a mutation in the *PIK3CA* gene

**DOI:** 10.1007/s10072-023-06898-3

**Published:** 2023-07-12

**Authors:** Antonella Costagliola, Felice Arcuri, Pietro Pelliccioni, Alessandro Malandrini, Nicola De Stefano, Carla Battisti

**Affiliations:** 1 Department of Medicine, Surgery and Neuroscience, University of Siena, Siena, Italy; 2 Department of Molecular and Developmental Medicine, University of Siena, Siena, Italy

## To the Editor-in-Chief

Fibro-adipose vascular anomaly (FAVA) syndrome, first described by Alomari in 2014, is an extremely rare disease (about 20 cases reported in the literature) characterized by fibrofatty infiltration of skeletal muscle (mainly in the lower extremities) and unusual phlebectasia with pain and contracture of the affected extremity [1]. It usually occurs in young females (median age 12–17 years) but has also been described at birth or in early adulthood [2]. FAVA belongs to the spectrum of phosphatidylinositol-4,5-bisphosphate 3-kinase (PIK3CA)-related overgrowth syndromes (PROS); in most patients, it is caused by a somatic gene mutation. The gene activates the mammalian target of rapamycin (mTOR) pathway, known to promote angiogenesis and lymphangiogenesis [3]. Here, we describe an unusual new case of FAVA syndrome.

## Case report

**Fig. 1:**
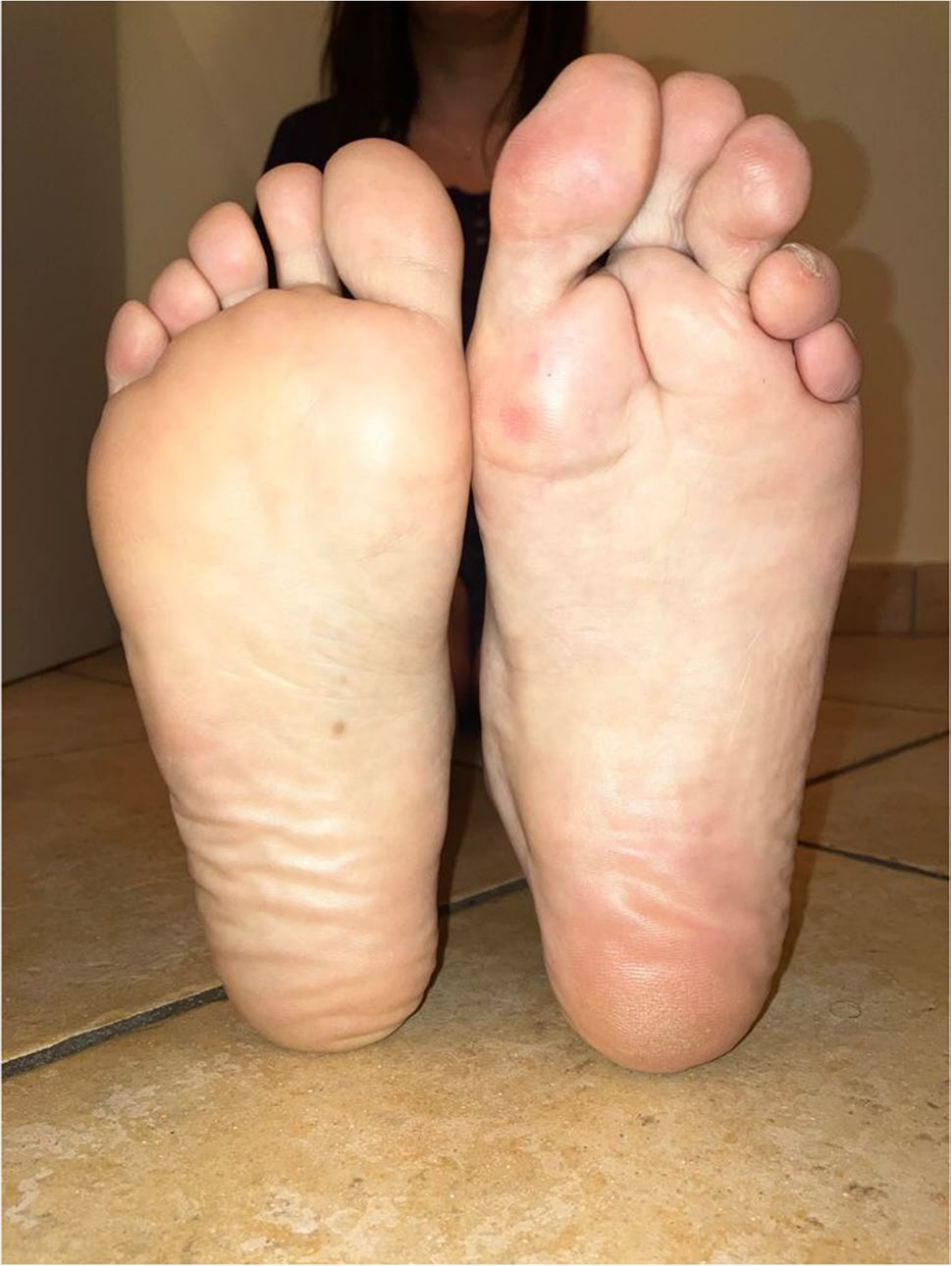
Patient’s feet showing overgrowth and foot drop of the left lower limb

A 34-year-old woman was referred to our unit in January 2022; her family and medical history were unremarkable. Neurological examination showed overgrowth and weakness of the left lower limb with left foot drop and gait imbalance (Fig. 1). The physical abnormalities were already present at birth when the patient was treated with a cast brace for the foot abnormality for 6 months. Weakness progressed very slowly over the years. The patient became a paralympic athlete, participating in world-class events until forced to quit at age 30 years due to a significant deterioration in her physical performance. She underwent a first electroneuromyography of the lower limbs at age 20, which showed neurotmesis of the left external popliteal nerve at the fibular head. At age 32, a musculoskeletal ultrasound (US) of the lower limbs showed bilateral fibrous infiltration of the biceps femoris, medial gastrocnemius, tibialis posterior, and fibularis longus; severe alterations were detected by a pelvic MRI in the same period (muscle atrophy with diffuse fatty infiltration of the gluteus minimus, gluteus medius, iliac, and vastus lateralis). Another electroneuromyography of the lower limbs performed 2 years later showed integrity of the left peroneus communis (the longest fibers innervated the extensor digitorum brevis) and diffuse denervation of all other muscles tested (tibialis anterior, fibularis longus, and medial gastrocnemius). Doppler US of lower limbs and abdominal US were both normal. Genetic testing for *PIK3CA* mutations, performed elsewhere on the saliva, skin, and blood samples, was negative.

**Fig. 2:**
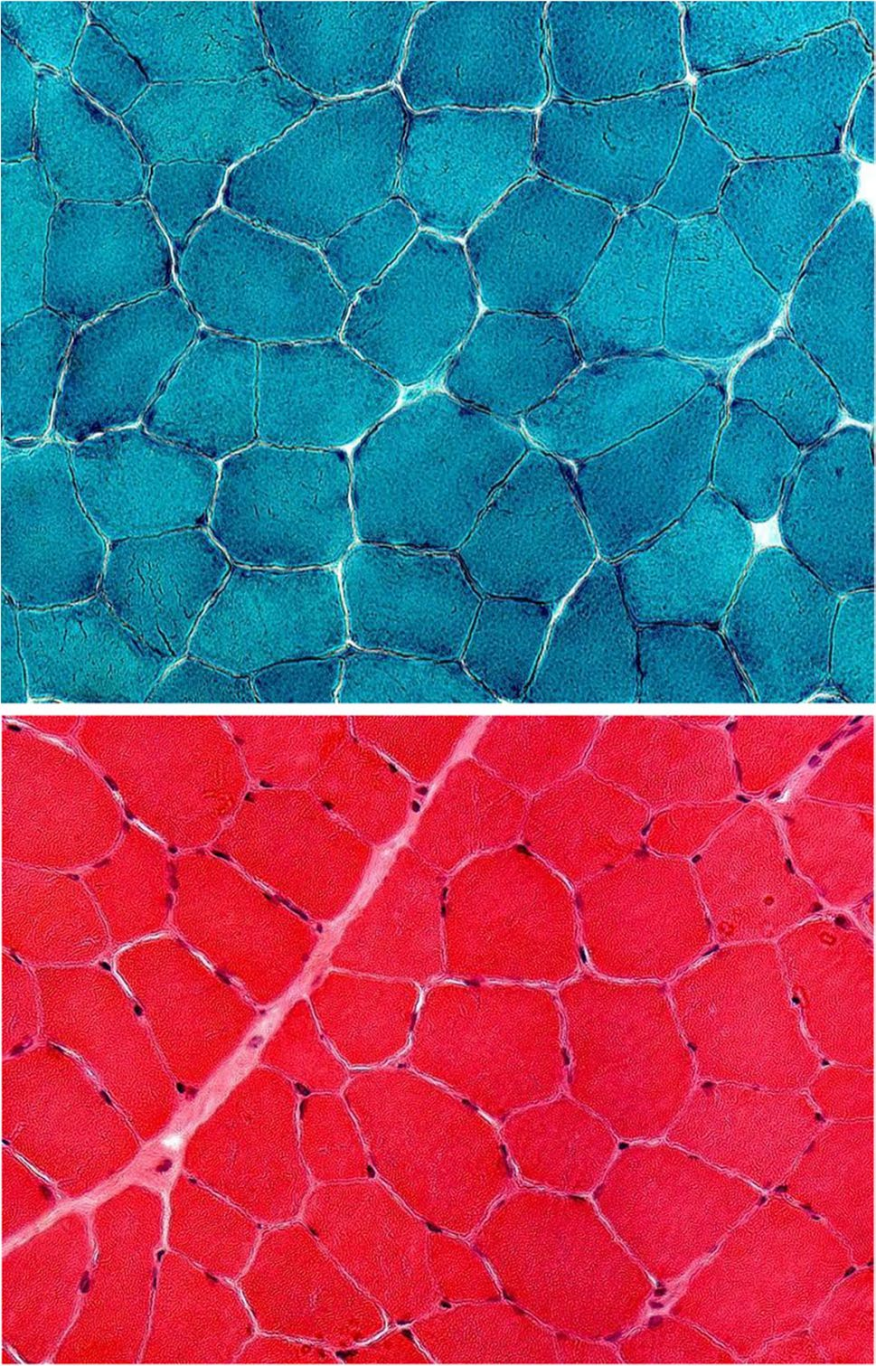
Muscle biopsy of the left vastus lateralis with hematoxylin–eosin and Gomori trichrome staining (magnification 250 ×): slight variation in fiber size

A muscle biopsy of the left vastus lateralis did not show histological evidence of fibrofatty infiltration (Fig. 2).

Genetic testing for *PIK3CA* mutations was negative on the saliva, skin, and blood samples, but on the muscle sample, we detected a variation in the *PIK3CA* gene, namely, a missense mutation at codon 542 [c.1624G > A p.(E542K)].

**Fig. 3:**
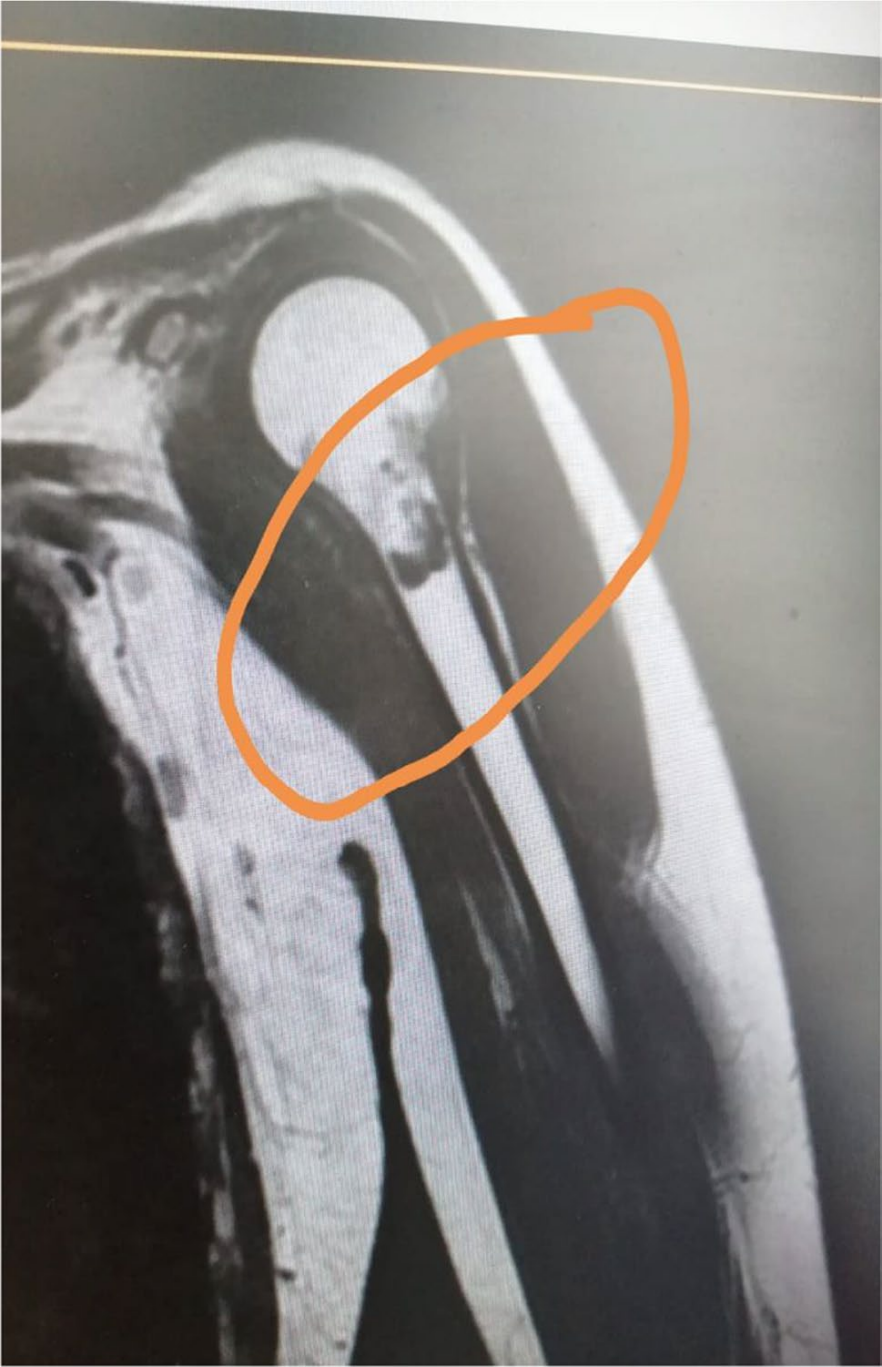
T1-weighted MRI following contrast administration showing on the left arm a chondroma of humerus (circle)

The MRI of the shoulders (June 2022) showed fatty infiltration of the deltoid muscle and a metaphyseal chondroma affecting the left humerus (Fig. 3).

An abdominal US performed in July 2022 showed bilateral fatty infiltration of the rectus abdominis. In August 2022, the patient complained of pain in the left index finger, due to a bony calcification in the synovial capsule of the same finger.

Since the 1st of February 2023, the patient has been treated with alpelisib, the first approved treatment to specifically address the pathogenic mechanisms involved in PROS in patients ≥ 2 years of age.

## Discussion

In the 2018 revision of the International Society for the Study of Vascular Anomalies (ISSVA) classification, FAVA syndrome was included in the provisionally “unclassified vascular anomalies” category.

FAVA mainly affects young females (median age, 12–17 years) but has been described from birth to early adulthood. The syndrome generally presents with functional restrictions (reported in 81% of patients), local pain (100% patients), and swelling (62% patients) [4]. Lower limbs are often involved early in the course of the disease, the gastrocnemius–soleus muscle group being the most affected. Many patients with calf involvement have limited ankle dorsiflexion (equinus foot). Most patients are initially prescribed orthotics to correct limb malposition. Upper limb lesions are mainly reported in the flexor compartment of the forearm, associated with extension into the carpal tunnel and proximal palm.

Histologically, FAVA is characterized by venous malformations (VM), lymphatic malformations (LM), and fibro-adipose infiltration of the skeletal muscle [3], the latter only occurring in areas with histological evidence of microvascular malformations. The degree of fibrofatty infiltration of muscle varies from focal lesions in isolated muscles to widespread involvement of the flexor-pronator compartment.

Two main features differentiate FAVA from other combined vascular malformations, such as intramuscular vascular malformations (e.g., cystic lymphatic and vascular malformations, Klippel–Trenaunay syndrome, congenital lipomatous overgrowth, epidermal nevi): (1) abnormal veins are often thin walled and dysplastic, and (2) the fibrotic tissue may encase neurovascular structures causing compression symptoms.

Since the syndrome is usually diagnosed late, often due to overlap of clinical features with other common entities, symptoms and signs are frequently severe at diagnosis, typically featuring increased contracture formation and intractable pain [5].

Treatment options for FAVA syndrome include cryoablation, surgery, and pharmacotherapy. Sclerotherapy with sodium tetradecyl sulfate (STS) or bleomycin is offered as first-line treatment especially in cases with Doppler US evidence of a significant vascular component. Cryoablation is used in selected cases with a solid component, producing good patient-reported outcomes at 2–5-month follow-up but unclear long-term benefit. The indication for surgical treatment is intractable pain and/or contractures leading to significant loss of function and reduced quality of life. All surgical approaches are aimed at removing lesions while preserving normal structures and restoring function by correcting tendon and joint contractions [4]. Finally, pharmacological options include the mTOR inhibitor sirolimus and PIK3 inhibitor alpelisib, which have shown high tolerability and effectiveness. Initial symptom improvement is rapid, with significantly reduced pain and improved function. These two options should therefore be considered as first-line therapy for all patients with FAVA.

The new case of FAVA syndrome reported here has some significant atypical features: (1) the discrepancy between the slow progression of motor function impairment prior to age 30 years and the relatively rapid progression of muscle atrophy: unlike the other cases described in the literature, our patient had mild symptoms until her early 30 s and did not require surgery [6]; (2) the absence of histological evidence of fibrofatty infiltration of muscle, despite a genetic variation in the *PIK3CA* gene, suggesting that selection of the biopsy site is of paramount importance (only muscle near ectatic vessels shows fibrofatty infiltration).

In conclusion, FAVA syndrome should be considered in differential diagnosis of vascular abnormalities of the lower limbs. Negative biopsy results should not discourage from proceeding with genetic testing if clinical features support the suspect of FAVA syndrome.

## Data Availability

Written consent was signed by the patient before the drafting of this article.
